# Lines on Indigenous Medicinal Plants

**Published:** 1866-09

**Authors:** D. L. Phares

**Affiliations:** Newtonia, Mississippi


ARTICLE II.

*Lines on Indigenous Medicinal Plants.* By D. L. PHARES, M. D., Newtonia, Mississippi.

Every physician should be well versed in botany, so as to be able to distinguish properly and recognize all plants coming under his observation. But botany is sadly neglected in all the schools. Few physicians have had the opportunity of attaining to any considerable proficiency in this science, which presents for our consideration so many beauties, odors, flavors and adaptations, addressed to all our senses as well as to our reason. These beauties, adaptations and blessed properties are literally thrust upon us at every step, whether in field or forest, mountain brow or water-covered vale. We dash them aside without reflection; we cast aside as useless the healing balm, the life-giving plant, beneficently prepared by the All-Wise Physician for the relief of human suffering and woe.

But disliking prolixity, circumlocution, and useless verbiage, which no one has a right to inflict on another, I proceed at once and with as much brevity as possible to present to my medical brethren a few valuable remedies derived from our Southern Flora—some of them now for the first time offered to the profession. By way of introducing another agent, I will say a word in regard to the

*Dioscorea Villosa.*—Although my ancient friend, the late learned and scientific Prof. Riddell, of the University of Louisiana, made known the properties of this plant some thirty-five years ago, few of the regular profession seem to be aware of its value, or even of its existence. Griffith, in his "Medical Botany," quoting Riddell, disposes of it in four brief sentences. It grows on the margins of swamps in all the States. The introduced species, D. *sativa* and *alata*, have been long cultivated on the gulf coast, for their large edible tubers. The D. *batatas* was brought from China, by way of France, to the United States in 1854–5, and is now cultivated for culinary purposes in all the States to a limited extent. Our native species, however, is the only one valuable as a medicine—the contorted, knotty root being the part used.

It is diaphoretic, expectorant and antispasmodic, with special direction to the stomach and bowels. It has other adaptations; but I commend it specially as a specific for bilious colic. However violent the attack, in whatever stage, even though chloroform, salts of morphia and other remedies produce no perceptible effect, this remedy relieves invariably, promptly. Whether used after other remedies had failed, or alone, even in the most desperate cases, I have seen a single pill of the extract, in less than five minutes, produce profound but natural sleep, from which the patient awoke *well!* This I have seen many times. The prompt, complete, permanent relief it affords is truly astonishing. For colic, it is more potent than quinine for intermittent, morphine for pain, or chloroform for spasm. A fluid extract, which I have never used, is prepared by some of the pharmaceutists. They also make a dry pulveralent extract under the name of dioscorcine, most of which is worthless. I received some years ago a very superior article of dioscorcine from New York—the dose being from three to five grains. A strong decoction of the root may be given in doses of four to eight ounces. This medicine is much used by a certain sect, whose indefatigable industry and energy in exploring and developing the varied and rich resources of our indigenous medical flora is worthy of commendation and emulation by the members of our noble and honorable fraternity.

Some years ago, unable to get a reliable extract, I wished to obtain the dioscorea in a recent state. I described it to several persons, requesting them to find it for me. One brought me an imperfect specimen of what he said his father (a very intelligent farmer) called "colic vine," and had used for many years in cases of colic. Some fifteen months after this, I obtained perfect plants and found it to be, (what I had already conjectured from its family likeness,)

*Desmodium Rotundifolium.*—I have used this plant, both in recent and dry state, leaves and stems, in many cases of colic in subjects of all ages and conditions, with complete success. I have never used it, however, in any very severe cases of bilious colic. It is probable that a concentrated extract might effect as much in this distressing complaint as that of dioscorea. It is a most admirable thing in infantile colics and dysentery, being pleasant to take, affording prompt relief, and having no narcotic or other deleterious properties. It is nervine, tonic antispasmodic, slightly astringent, acting mainly on the abdominal and pelvic viscera, relieving colic, cramp, spasm, tenesmus, &c. The tea may be taken *ad libitum.* The small tubes attached to the roots may prove as valuable as the leaf. I have not tried them. This plant grows in dry, open woods throughout the Southern States, that on thin, close soil being best.

Several other species, as the D. acuminatum, and perhaps a species or two of the allied genus, lespedeza, I am persuaded, possess very valuable medicinal properties and are worthy of examination. None of them have ever been, so far as I know, heretofore recognized or described as medicinal.

*Polymnia Uvedalia.*—This plant may be found growing on rich soil in all the Southern States; some farmers protecting and encouraging its propagation. The root is the most valuable part, being sliced or bruised and simmered in lard for local application, or made into a saturated tincture for internal use. It is a valuable remedy in scrofulous tumors and ulcers, both of the soft parts and bony structures, under whatever name described, as hip-joint, knee-joint, or heel disease, rickets, king's evil, tubercular deposit, &c. It has effected many cures in these affections, when other remedies have failed.

*Chrysopsis Graminifolia.*—(C. argentea, Nutt.)—Some years ago a party of Indians encamped for some weeks near the residence of Major Jones of this county, and having received kind treatment at his hands, pointed out to him several plants, whose virtues they made known to him. One of them has now been used by the Major and other citizens with such good results in so many cases of scrofulous affection, that it has become considerably known over a wide section of country by the name of "scrofula grass." On examination of the plant, I found it to be that named above. It is found in sandy, pine barrens, in all the Southern States, flowering in September and October, and in some localities very abundant.

The whole plant is used in watery infusion, or tea, and has effected many cures of scrofulous tumors and ulcers, and cutaneous eruptions. Some of the worst, most hopeless, disgusting and horrible cases of constitutional syphilis and mercurio-syphilitic diseases have been cured by this valuable agent. It is an alterant of extraordinary power, acting gently, unostentatiously, pleasantly, effectually. I have used neither of the last two articles to the extent which their great curative powers would warrant, as I had acquired a habit of using other indigenous agents, with whose effects I had been satisfied, in eliminating the syphilitic taint, renovating the blood, and reconstructing the new man from the old wreck. I shall, however, in future, avail more of their valuable properties, which are very decided. These brief notes are hurriedly written, in order to place the articles mentioned before the profession in time for them to try them this season.

